# Two new species of *Diaporthe* (*Diaporthaceae*, *Diaporthales*) associated with tree cankers in ﻿the Netherlands

**DOI:** 10.3897/mycokeys.85.73107

**Published:** 2021-11-29

**Authors:** Ning Jiang, Hermann Voglmayr, Chun-Gen Piao, Yong Li

**Affiliations:** 1 Key Laboratory of Forest Protection of National Forestry and Grassland Administration, Institute of Forest Ecology, Environment and Nature Conservation, Chinese Academy of Forestry, Beijing 100091, China Environment and Nature Conservation, Chinese Academy of Forestry Beijing China; 2 Department of Botany and Biodiversity Research, University of Vienna, Rennweg 14, A-1030 Vienna, Austria Beijing Forestry University Beijing China; 3 The Key Laboratory for Silviculture and Conservation of the Ministry of Education, Beijing Forestry University, Beijing 100083, China University of Vienna Vienna Austria

**Keywords:** Two new taxa, *Diaporthepseudoalnea*, *Diaporthesilvicola*, taxonomy, two new taxa

## Abstract

*Diaporthe* (*Diaporthaceae*, *Diaporthales*) is a common fungal genus inhabiting plant tissues as endophytes, pathogens and saprobes. Some species are reported from tree branches associated with canker diseases. In the present study, *Diaporthe* samples were collected from *Alnusglutinosa*, *Fraxinusexcelsior* and *Quercusrobur* in Utrecht, the Netherlands. They were identified to species based on a polyphasic approach including morphology, pure culture characters, and phylogenetic analyses of a combined matrix of partial ITS, *cal*, *his3*, *tef1* and *tub2* gene regions. As a result, four species (viz. ﻿﻿***Diaporthepseudoalnea* sp. nov.** from *Alnusglutinosa*, ***Diaporthesilvicola* sp. nov.** from *Fraxinusexcelsior*, *D.foeniculacea* and *D.rudis* from *Quercusrobur*) were revealed from tree branches in the Netherlands. *Diaporthepseudoalnea* differs from *D.eres* (syn. *D.alnea*) by its longer conidiophores. *Diaporthesilvicola* is distinguished from *D.fraxinicola* and *D.fraxini-angustifoliae* by larger alpha conidia.

## ﻿Introduction

*Diaporthe* (syn. *Phomopsis*) is the type genus of *Diaporthaceae* in *Diaporthales*, commonly occurring as plant endophytes, pathogens and ﻿saprobes (Udayanga et al. 2014, 2015; Guarnaccia et al. 2017, 2018a, 2018b; Tibpromma et al. 2018; Yang et al. 2020; Dissanayake et al. 2020; Jiang et al. 2021). The sexual morph is characterized by immersed perithecial ascomata and an erumpent pseudostroma with more or less elongated perithecial necks, unitunicate clavate to cylindrical asci, and fusoid, ellipsoid to cylindrical, hyaline uni- to bicellular ascospores (Udayanga et al. 2011; Senanayake et al. 2017). The asexual morph is characterized by ostiolate conidiomata, with cylindrical phialides producing up to three types of hyaline, aseptate conidia (Udayanga et al. 2011; Gomes et al. 2013; Yang et al. 2018), and was previously classified as *Phomopsis*. Following the “one fungus one name” nomenclature, Rossman et al. (2015) recommended to use *Diaporthe* based on priority, necessitating the transfer of numerous *Phomopsis* species to *Diaporthe*.

Species of *Diaporthe* are known to cause plant diseases including dieback, canker, leaf spot, fruit rot, pod blights and seed decay. For example, *D.citri*, *D.cytosporella* and *D.foeniculina* caused melanose and stem end rot diseases of *Citrus* spp. (Udayanga et al. 2014), while *Daporthelithocarpi* caused leaf spot disease of *Castaneahenryi* in China (Jiang et al. 2021﻿). Up to 19 *Diaporthe* species were confirmed to be associated with pear cankers in China (Guo et al. 2020), and eight species of *Diaporthe* were found to be the casual agents of Chinese grapevine dieback (Manawasinghe et al. 2019). Seven *Diaporthe* species were reported from blueberry twig blight and dieback diseases in Portugal (Hilário et al. 2020). *Diaporthebiconispora* and an additional six species were identified as endophytes from healthy *Citrus* tissues in China (Huang et al. 2015). *Diaportheconstrictospora* and an additional 11 species were isolated as saprobes from dead wood in karst formations in China (Dissanayake et al. 2020).

*Diaporthe* species were previously classified mainly based on host association and morphology (Rehner and Uecker 1994; Santos and Phillips 2009﻿; Udayanga et al. 2011, 2014). However, several taxonomic studies of *Diaporthales* proved that phylogeny based on multiple genes is suitable to separate species (Voglmayr et al. 2012, 2017; Fan et al. 2018; Jiang et al. 2019, 2020; Jaklitsch and Voglmayr 2019, 2020). Species of *Diaporthe* are now characterised and circumscribed both by morphology and phylogeny of multi-locus DNA data, which revealed many cryptic species in recent years (Diogo et al. 2010; Lombard et al. 2014; Gao et al. 2016, 2017; Long et al. 2019; Yang et al. 2020, 2021; Zapata et al. 2020; Huang et al. 2021). To clarify the species boundaries of the *Diaportheeres* complex, the Genealogical Phylogenetic Species Recognition principle (GCPSR) and the coalescent-based model Poisson Tree Processes (PTPs) were employed, which suggested that the *Diaportheeres* species complex actually represents only a single species, *D.eres* (Hilário et al. 2021).

In the present study, *Diaporthe* samples from cankered branches of several tree species were collected in the Netherlands, and identified based on modern taxonomic approaches. As a result, two new species and two known species were identified, and the new species are described and illustrated herein.

## Materials and Methods

### Collection, examination and isolation

The fresh specimens of cankered branches were sampled from *Alnusglutinosa*, *Fraxinusexcelsior* and *Quercusrobur* in Utrecht, the Netherlands. Morphological characteristics of the conidiomata were determined under a Nikon AZ100 dissecting stereomicroscope. More than 20 conidiomata were sectioned, and 50 conidia were randomly selected for measurement using a Leica compound microscope (LM, DM 2500). Isolates were obtained by removing a mucoid conidial mass from conidiomata, spreading the suspension onto the surface of 1.8 % potato dextrose agar (PDA), and incubated at 25 °C for up to 24 h. Single germinating conidia were removed and plated onto fresh PDA plates. Cultural characteristics of isolates incubated on PDA in the dark at 25 °C were recorded, including the colony color and conidiomata structures. The cultures were deposited in the China Forestry Culture Collection Center (CFCC; http://www.cfcc-caf.org.cn/), and the specimens in the herbarium of the Chinese Academy of Forestry (CAF; http://museum.caf.ac.cn/).

### DNA extraction, PCR amplification and phylogenetic analyses

Genomic DNA was extracted from colonies grown on cellophane-covered PDA using a cetyltrimethylammonium bromide (CTAB) method (Doyle and Doyle 1990). DNA was checked by electrophoresis in 1 % agarose gel, and the quality and quantity were measured using a NanoDrop 2000 (Thermo Scientific, Waltham, MA, USA). Five partial loci, including the 5.8S nuclear ribosomal DNA gene with the two flanking internally transcribed spacer (ITS) regions, the calmodulin (*cal*), the histone H3 (*his3*), the translation elongation factor 1-alpha (*tef1*) and the beta-tubulin (*tub2*) genes were amplified by the primer pairs and polymerase chain reaction (PCR) process listed in Table 1. The PCR products were assayed via electrophoresis in 2 % agarose gels. DNA sequencing was performed using an ABI PRISM 3730XL DNA Analyser with a BigDye Terminator Kit v.3.1 (Invitrogen, USA) at the Shanghai Invitrogen Biological Technology Company Limited (Beijing, China).

**Table 1.** Genes used in this study with PCR primers and process.

**Locus**	**PCR primers**	**PCR: thermal cycles: (Annealing temp. in bold)**	**Reference**
ITS	ITS1/ITS4	(95 °C: 30 s, **48 °C**: 30 s, 72 °C: 1 min) × 35 cycles	White et al. 1990
*cal*	CAL228F/CAL737R	(95 °C: 15 s, **54 °C**: 20 s, 72 °C: 1 min) × 35 cycles	Carbone and Kohn 1999
*his3*	CYLH3F/H3-1b	(95 °C: 30 s, **57 °C**: 30 s, 72 °C: 1 min) × 35 cycles	Crous et al. 2004 Glass and Donaldson 1995
*tef1*	EF1-728F/EF1-986R	(95 °C: 15 s, **54 °C**: 20 s, 72 °C: 1 min) × 35 cycles	Carbone and Kohn 1999
*tub2*	T1(Bt2a)/Bt2b	(95 °C: 30 s, **55 °C**: 30 s, 72 °C: 1 min) × 35 cycles	Glass & Donaldson 1995; O’Donnell and Cigelnik 1997

The quality of the amplified nucleotide sequences was checked and the sequences assembled using SeqMan v.7.1.0. Reference sequences were retrieved from the National Center for Biotechnology Information (NCBI), based on recent publications on the genus *Diaporthe* (Dissanayake et al. 2021; Gao et al. 2021; Huang et al. 2021; Sun et al. 2021, Wang et al. 2021; Yang et al. 2021). Sequences were aligned using MAFFT v. 6 (Katoh and Toh 2010) and corrected manually using MEGA 7.0.21. The best-fit nucleotide substitution models for each gene were selected using jModelTest v. 2.1.7 (Darriba et al. 2012) under the Akaike Information Criterion.

The phylogenetic analyses of the combined gene regions were performed using Maximum Likelihood (ML) and Bayesian Inference (BI) methods. ML was implemented on the CIPRES Science Gateway portal (https://www.phylo.org) using RAxML-HPC BlackBox 8.2.10 (Stamatakis 2014), employing a GTRGAMMA substitution model with 1000 bootstrap replicates. While BI was performed using a Markov Chain Monte Carlo (MCMC) algorithm in MrBayes v. 3.0 (Ronquist et al. 2003). Two MCMC chains, started from random trees for 1000000 generations and trees, were sampled every 100th generation, resulting in a total of 10000 trees. The first 25 % of trees were discarded as burn-in of each analysis. Branches with significant Bayesian Posterior Probabilities (BPP) were estimated in the remaining 7500 trees. Phylogenetic trees were viewed with FigTree v.1.3.1 and processed by Adobe Illustrator CS5. The nucleotide sequence data of the new taxa were deposited in GenBank and are listed in Table 2.

**Table 2.** Isolates and GenBank accession numbers used in the phylogenetic analyses of *Diaporthe*.

Species	Strain	Host	Origin	GenBank accession numbers				
				ITS	*cal*	*his3*	*tef1*	*tub2*
*Diaportheacaciigena*	CBS 129521	*Acaciaretinodes*	Australia	KC343005	KC343247	KC343489	KC343731	KC343973
*D.acericola*	MFLUCC 17-0956	*Acernegundo*	Italy	KY964224	KY964137	NA	KY964180	KY964074
*D.acerigena*	CFCC 52554	*Acertataricum*	China	MH121489	MH121413	MH121449	MH121531	NA
*D.acerigena*	CFCC 52555	*Acertataricum*	China	MH121490	MH121414	MH121450	MH121532	NA
*D.acuta*	PSCG 047	*Pyruspyrifolia*	China	MK626957	MK691125	MK726161	MK654802	MK691225
*D.acutispora*	LC6161	*Coffea*	China	KX986764	KX999274	KX999235	KX999155	KX999195
*D.alangii*	CFCC 52556	*Alangiumkurzii*	China	MH121491	MH121415	MH121451	MH121533	MH121573
*D.alangii*	CFCC 52557	*Alangiumkurzii*	China	MH121492	MH121416	MH121452	MH121534	MH121574
*D.albosinensis*	CFCC 53066	*Betulaalbosinensis*	China	MK432659	MK442979	MK443004	MK578133	MK578059
*D.albosinensis*	CFCC 53067	*Betulaalbosinensis*	China	MK432660	MK442980	MK443005	MK578134	MK578060
*D.alleghaniensis*	CBS 495.72	*Betulaalleghaniensis*	Canada	MH121502	MH121426	MH121462	MH121544	MH121584
*D.ambigua*	CBS 114015	*Pyruscommunis*	South Africa	KC343010	KC343252	KC343494	KC343736	KC343978
*D.ampelina*	STE-U 2660	*Vitisvinifera*	France	NA	AY745026	NA	AY745056	NA
*D.amygdali*	CBS 126679	*Prunusdulcis*	Portugal	MH864208	KC343264	KC343506	KC343748	KC343990
*D.anacardii*	CBS 720.97	*Anacardiumoccidentale*	East Africa	KC343024	KC343266	KC343508	KC343750	KC343992
*D.angelicae*	CBS 111592	*Heracleumsphondylium*	Austria	KC343027	KC343269	KC343511	KC343753	KC343995
*D.apiculatum*	CFCC 53068	*Rhuschinensis*	China	MK432651	MK442973	MK442998	MK578127	MK578054
*D.apiculatum*	CFCC 53069	*Rhuschinensis*	China	MK432652	MK44297	MK442999	MK578128	MK578055
*D.aquatica*	IFRDCC 3051	*Aquatic habitat*	China	JQ797437	NA	NA	NA	NA
*D.arctii*	DP0482	*Arctiumlappa*	Austria	KJ590736	KJ612133	KJ659218	KJ590776	KJ610891
*D.arecae*	CBS 161.64	*Arecacatechu*	India	KC343032	KC343274	KC343516	KC343758	KC344000
*D.arengae*	CBS 114979	*Arengaengleri*	Hong Kong	MF773664	KC343276	KC343518	KC343760	KC344002
*D.aseana*	MFLUCC 12-0299a	Unknown	Thailand	KT459414	KT459464	NA	KT459448	KT459432
*D.asheicola*	CBS 136967	*Vacciniumashei*	Chile	KJ160562	KJ160542	NA	KJ160594	KJ160518
*D.aspalathi*	CBS 117169	*Aspalathuslinearis*	South Africa	KC343036	KC343278	KC343520	KC343762	KC344004
*D.australafricana*	CBS 111886	*Vitisvinifera*	Australia	KC343038	KC343280	KC343522	KC343764	KC344006
*D.australiana*	CBS 146457	*Macadamia*	Australia	MN708222	NA	NA	MN696522	MN696530
*D.baccae*	CBS 136972	*Vacciniumcorymbosum*	Italy	MK370623	MG281695	MF418264	KJ160597	MF418509
*D.batatas*	CBS 122.21	*Ipomoeabatatas*	USA	KC343040	KC343282	KC343524	KC343766	KC344008
*D.bauhiniae*	CFCC 53071	*Bauhiniapurpurea*	China	MK432648	MK442970	MK442995	MK578124	MK578051
*D.bauhiniae*	CFCC 53072	*Bauhiniapurpurea*	China	MK432649	MK442971	MK442996	MK578125	MK578052
*D.bauhiniae*	CFCC 53073	*Bauhiniapurpurea*	China	MK432650	MK442972	MK442997	MK578126	MK578053
*D.beilharziae*	BRIP 54792	*Indigoferaaustralis*	Australia	JX862529	NA	NA	JX862535	KF170921
*D.benedicti*	SBen914	*Diaporthebenedicti*	USA	KM669929	KM669862	NA	KM669785	NA
*D.betulae*	CFCC 50469	*Betulaplatyphylla*	China	KT732950	KT732997	KT732999	KT733016	KT733020
*D.betulae*	CFCC 50470	*Betulaplatyphylla*	China	KT732951	KT732998	KT733000	KT733017	KT733021
*D.betulicola*	CFCC 51128	*Betulaalbo-sinensis*	China	KX024653	KX024659	KX024661	KX024655	KX024657
*D.betulicola*	CFCC 51129	*Betulaalbo-sinensis*	China	KX0246554	KX024660	KX024662	KX0246556	KX024658
*D.betulina*	CFCC 52560	*Betulaalbo-sinensis*	China	MH121495	MH121419	MH121455	MH121537	MH121577
*D.betulina*	CFCC 52561	*Betulaalbo-sinensis*	China	MH121496	MH121420	MH121456	MH121538	MH121578
*D.biconispora*	ZJUD62	*Citrusmaxima*	China	KJ490597	NA	KJ490539	KJ490476	KJ490418
*D.biguttulata*	ZJUD47	*Citruslimon*	China	KJ490582	NA	KJ490524	KJ490461	KJ490403
*D.bohemiae*	CBS 143347	*Vitisvinifera*	Czech Republic	MK300012	MG281710	MG281361	MG281536	MG281188
*D.brasiliensis*	CBS 133183	*Aspidospermatomentosum*	Brazil	KC343042	KC343284	KC343526	KC343768	KC344010
*D.caatingaensis*	URM7485	*Tacingainamoena*	Brazil	KY085927	KY115598	NA	KY115604	KY115601
*D.camelliae-sinensis*	SAUCC194.92	*Camelliasinensis*	China	MT822620	MT855699	MT855588	MT855932	MT855817
*D.canthii*	CPC 19740	*Canthiuminerme*	South Africa	JX069864	NA	NA	NA	NA
*D.caryae*	CFCC 52563	*Caryaillinoinensis*	China	MH121498	MH121422	MH121458	MH121540	MH121580
*D.caryae*	CFCC 52564	*Caryaillinoinensis*	China	MH121499	MH121423	MH121459	MH121541	MH121581
*D.cassines*	CPC 21916	*Cassineperagua*	South Africa	KF777155	NA	NA	KF777244	NA
*D.caulivora*	CBS 127268	*Glycinemax*	Croatia	MH864501	KC343287	KC343529	KC343771	KC344013
*D.cercidis*	CFCC 52565	*Cercischinensis*	China	MH121500	MH121424	MH121460	NA	MH121582
*D.cercidis*	CFCC 52566	*Cercischinensis*	China	MH121501	MH121425	MH121461	NA	MH121583
*D.chamaeropis*	CBS 454.81	*Chamaeropshumilis*	Greece	KC343048	KC343290	KC343532	KC343774	KC344016
*D.charlesworthii*	BRIP 54884m	*Rapistrumrugostrum*	Australia	KJ197288	NA	NA	KJ197250	KJ197268
*D.chensiensis*	CFCC 52567	*Abieschensiensis*	China	MH121502	MH121426	MH121462	MH121544	MH121584
*D.chensiensis*	CFCC 52568	*Abieschensiensis*	China	MH121503	MH121427	MH121463	MH121545	MH121585
*D.chongqingensis*	PSCG 435	*Pyruspyrifolia*	China	MK626916	MK691209	MK726257	MK654866	MK691321
*D.chrysalidocarpi*	SAUCC194.35	*Chrysalidocarpuslutescens*	China	MT822563	MT855646	MT855532	MT855760	MT855876
*D.cichorii*	MFLUCC 17-1023	*Cichoriumintybus*	Italy	KY964220	KY964133	NA	KY964176	KY964104
*D.cinnamomi*	CFCC 52569	*Cinnamomum*	China	MH121504	NA	MH121464	MH121546	MH121586
*D.cinnamomi*	CFCC 52570	*Cinnamomum*	China	MH121505	NA	MH121465	MH121547	MH121587
*D.cissampeli*	CPC 27302	*Cissampeloscapensis*	South Africa	KX228273	NA	KX228366	NA	KX228384
*D.citri*	AR3405	*Citrus*	USA	KC843311	KC843157	KJ420881	KC843071	KC843187
*D.citri*	CFCC 53079	*Citrussinensis*	China	MK573940	MK574579	MK574595	MK574615	MK574635
*D.citriasiana*	CGMCC 3.15224	*Citrusunshiu*	China	JQ954645	KC357491	KC490515	JQ954663	KC357459
*D.citrichinensis*	CGMCC 3.15225	*Citrus*	China	JQ954648	KC357494	NA	JQ954666	NA
*D.collariana*	MFLU 17-2770	*Magnoliachampaca*	Thailand	MG806115	MG783042	NA	MG783040	MG783041
*D.compactum*	LC3083	*Camelliasinensis*	China	KP267854	NA	KP293508	KP267928	NA
*D.conica*	CFCC 52571	*Alangiumchinense*	China	MH121506	MH121428	MH121466	MH121548	MH121588
*D.conica*	CFCC 52572	*Alangiumchinense*	China	MH121507	MH121429	MH121467	MH121549	MH121589
*D.constrictospora*	CGMCC 3.20096	Unknown	China	MT385947	MT424718	MW022487	MT424682	MT424702
*D.convolvuli*	CBS 124654	*Convolvulusarvensis*	Turkey	KC343054	KC343296	KC343538	KC343780	KC344022
*D.coryli*	CFCC 53083	*Corylusmandshurica*	China	MK432661	MK442981	MK443006	MK578135	MK578061
*D.coryli*	CFCC 53084	*Corylusmandshurica*	China	MK432662	MK442982	MK443007	MK538176	MK578062
*D.corylicola*	CFCC 53986	*Corylusheterophylla*	China	MW839880	MW836684	MW836717	MW815894	MW883977
*D.corylicola*	CFCC 53987	*Corylusheterophylla*	China	MW839867	MW836685	MW836718	MW815895	MW883978
*D.crotalariae*	CBS 162.33	*Crotalariaspectabilis*	USA	MH855395	JX197439	KC343540	GQ250307	KC344024
*D.crousii*	CAA 823	*Vacciniumcorymbosum*	Portugal	MK792311	MK883835	MK871450	MK828081	MK837932
*D.cucurbitae*	DAOM 42078	*Cucumis*	Canada	KM453210	NA	KM453212	KM453211	KP118848
*D.cuppatea*	CBS 117499	*Aspalathuslinearis*	South Africa	MH863021	KC343299	KC343541	KC343783	KC344025
*D.cynaroidis*	CBS 122676	*Proteacynaroides*	South Africa	KC343058	KC343300	KC343542	KC343784	KC344026
*D.cytosporella*	FAU461	*Citruslimon*	Italy	KC843307	KC843141	NA	KC843116	KC843221
*D.diospyricola*	CPC 21169	*Diospyroswhyteana*	South Africa	KF777209	NA	NA	NA	NA
*D.discoidispora*	ZJUD89	*Citrusunshiu*	China	KJ490624	NA	KJ490566	KJ490503	KJ490445
*D.dorycnii*	MFLUCC 17-1015	*Dorycniumhirsutum*	Italy	KY964215	NA	NA	KY964171	KY964099
*D.drenthii*	CBS 146453	*Macadamia*	Australia	MN708229	NA	NA	MN696526	MN696537
*D.elaeagni-glabrae*	LC4802	*Elaeagnusglabra*	China	KX986779	KX999281	KX999251	KX999171	KX999212
*D.ellipicola*	CGMCC 3.17084	*Lithocarpusglaber*	China	KF576270	NA	NA	KF576245	KF576294
*D.endophytica*	CBS 133811	*Schinusterebinthifolius*	Brazil	KC343065	KC343307	KC343549	KC343791	KC344033
*D.eres*	CBS 146.46	*Alnus*	Netherlands	KC343008	KC343250	KC343492	KC343734	KC343976
*D.eres*	CBS 121004	*Juglans*	USA	KC343134	KC343376	KC343618	KC343860	KC344102
*D.eres*	CGMCC 3.17081	*Lithocarpusglabra*	China	KF576282	NA	NA	KF576257	KF576306
*D.eres*	CFCC 51632	*Camptothecaacuminata*	China	KY203726	KY228877	KY228881	KY228887	KY228893
*D.eres*	CBS 139.27	*Celastrus*	USA	KC343047	KC343289	KC343531	KC343773	KC344015
*D.eres*	CBS 143349	*Vitisvinifera*	United Kingdom	MG281017	MG281712	MG281363	MG281538	MG281190
*D.eres*	AR5193	*Ulmus*	Germany	KJ210529	KJ434999	KJ420850	KJ210550	KJ420799
*D.eres*	CFCC 52575	*Castaneamollissima*	China	MH121510	NA	MH121470	MH121552	MH121592
*D.eres*	CFCC 52576	*Castaneamollissima*	China	MH121511	MH121432	MH121471	MH121553	MH121593
*D.eres*	CFCC 52577	*Acanthopanaxsenticosus*	China	MH121512	MH121433	MH121472	MH121554	MH121594
*D.eres*	CFCC 52578	*Sorbus*	China	MH121513	MH121433	MH121473	MH121555	MH121595
*D.eres*	CFCC 52579	*Juglansregia*	China	MH121514	NA	MH121474	MH121556	NA
*D.eres*	CFCC 52580	*Meliaazedarace*	China	MH121515	NA	MH121475	MH121557	MH121596
*D.eres*	CFCC 52581	*Rhododendronsimsii*	China	MH121516	NA	MH121476	MH121558	MH121597
*D.eres*	MAFF 625034	*Pyruspyrifolia*	Japan	NA	KJ435023	KJ420868	NA	KJ420819
*D.eres*	AR5211	*Hederahelix*	France	KJ210538	KJ435043	KJ420875	KJ210559	KJ420828
*D.eres*	CGMCC 3.17089	*Lithocarpusglabra*	China	KF576267	NA	NA	KF576242	KF576291
*D.eres*	MFLUCC 17-0963	*Lonicera*	Italy	KY964190	KY964116	NA	KY964146	KY964073
*D.eres*	DAOM 695742	*Picearuben*	Canada	KU552025	NA	NA	KU552023	KU574615
*D.eres*	MFLUCC 16-0113	*Prunuspersica*	China	KU557563	NA	KU557611	KU557631	KU55758
*D.eres*	CBS 144.27	*Spiraea*	USA	KC343144	KC343386	KC343628	KC343870	KC344112
*D.eres*	CBS 587.79	*Pinusparvifloravar*	Japan	KC343153	KC343395	KC343637	KC343879	KC344121
*D.eres*	CBS 338.89	*Hederahelix*	Yugoslavia	KC343152	KC343394	KC343636	KC343878	KC344120
*D.eres*	MFLU 17-0646	*Rosa*	United Kingdom	MG828895	MG829274	NA	MG829270	MG843877
*D.eucalyptorum*	CBS 132525	*Eucalyptus*	China	MH305525	NA	NA	NA	NA
*D.foeniculacea*	CBS 111553	*Foeniculumvulgare*	Spain	MH854926	KC343343	KC343585	KC343827	KC344069
***D.foeniculacea***	**CFCC 54192**	***Quercusrobur***	**Netherlands**	**MZ727033**	**NA**	**MZ753474**	**MZ816339**	**MZ753483**
***D.foeniculacea***	**M35**	***Quercusrobur***	**Netherlands**	**MZ727034**	**NA**	**MZ753475**	**MZ816340**	**MZ753484**
***D.foeniculacea***	**M40-1**	***Quercusrobur***	**Netherlands**	**MZ727035**	**NA**	**MZ753476**	**MZ816341**	**MZ753485**
***D.foeniculacea***	**M84**	***Quercusrobur***	**Netherlands**	**MZ727036**	**NA**	**MZ753477**	**MZ816342**	**MZ753486**
*D.fraxini-angustifoliae*	BRIP 54781	*Fraxinusangustifolia*	Australia	JX862528	KT459462	NA	JX862534	NA
*D.fraxinicola*	CFCC 52582	*Fraxinuschinensis*	China	MH121517	MH121435	NA	MH121560	NA
*D.fraxinicola*	CFCC 52583	*Fraxinuschinensis*	China	MH121518	MH121436	NA	MH121559	NA
*D.fulvicolor*	PSCG 051	*Pyruspyrifolia*	China	MK626859	MK691132	MK726163	MK654806	MK691236
*D.fusicola*	CGMCC 3.17087	*Lithocarpusglabra*	China	KF576281	KF576233	NA	KF576256	KF576305
*D.ganjae*	CBS 180.91	*Cannabissativa*	USA	KC343112	KC343354	KC343596	KC343838	KC344080
*D.ganzhouensis*	CFCC 53087	Unknown	China	MK432665	MK442985	MK443010	MK578139	MK578065
*D.ganzhouensis*	CFCC 53088	Unknown	China	MK432666	MK442986	MK443011	MK578140	MK578066
*D.garethjonesii*	MFLUCC 12-0542a	Unknown	Thailand	KT459423	KT459470	NA	KT459457	KT459441
*D.goulteri*	BRIP 55657a	*Helianthusannuus*	Australia	KJ197290	NA	NA	KJ197252	KJ197270
*D.grandiflori*	SAUCC194.84	*Heterostemmagrandiflorum*	China	MT822612	MT855691	MT855580	MT855809	MT855924
*D.guangxiensis*	JZB320087	*Vitisvinifera*	China	MK335765	MK736720	NA	MK500161	MK523560
*D.gulyae*	BRIP 54025	*Helianthusannuus*	Australia	NA	NA	NA	JN645803	KJ197271
*D.guttulata*	CGMCC 3.20100	Unknown	China	MT385950	MW022470	MW022491	MT424685	MT424705
*D.helianthi*	CBS 592.81	*Helianthusannuus*	Serbia	KC343115	KC343357	KC343599	KC343841	KC344083
*D.heliconiae*	SAUCC194.77	*Heliconiametallica*	China	MT822605	MT855684	MT855573	MT855802	MT855917
*D.heterophyllae*	CPC 26215	*Acaciaheterophylla*	France	MG600222	MG600218	MG600220	MG600224	MG600226
*D.heterostemmatis*	SAUCC194.85	*Heterostemmagrandiflorum*	China	MT822613	MT855692	MT855581	MT855810	MT855925
*D.hickoriae*	CBS 145.26	*Caryaglabra*	USA	KC343118	KC343360	NA	KC343844	KC344086
*D.hispaniae*	CBS 143351	*Vitisvinifera*	Spain	MG281123	MG281820	MG281471	MG281644	MG281296
*D.hongkongensis*	CBS 115448	*Dichroafebrifuga*	China	MK304388	KC343361	KC343603	KC343845	KC344087
*D.hubeiensis*	JZB320123	*Vitisvinifera*	China	MK335809	MK500235	NA	MK523570	MK500148
*D.incompleta*	LC6754	*Camelliasinensis*	China	KX986794	KX999289	KX999265	KX999186	KX999226
*D.inconspicua*	CBS 133813	*Maytenusilicifolia*	Brazil	NA	KC343365	KC343607	KC343849	KC344091
*D.infecunda*	CBS 133812	*Schinusterebinthifolius*	Brazil	KC343126	KC343368	KC343610	KC343852	KC344094
*D.irregularis*	CGMCC 3.20092	Unknown	China	MT385951	MT424721	NA	MT424686	MT424706
*D.isoberliniae*	CPC 22549	*Isoberliniaangolensis*	Zambia	KJ869190	NA	NA	NA	KJ869245
*D.juglandicola*	CFCC 51134	*Juglansmandshurica*	China	KU985101	KX024616	KX024622	KX024628	KX024634
*D.kadsurae*	CFCC 52586	*Kadsuralongipedunculata*	China	MH121521	MH121439	MH121479	MH121563	MH121600
*D.kadsurae*	CFCC 52587	*Kadsuralongipedunculata*	China	MH121522	MH121440	MH121480	MH121564	MH121601
*D.kochmanii*	BRIP 54033	*Helianthusannuus*	Australia	NA	NA	NA	JN645809	NA
*D.kongii*	BRIP 54031	*Helianthusannuus*	Australia	NA	NA	NA	NA	KJ197272
*D.lenispora*	CGMCC 3.20101	Unknown	China	MT385952	MW022472	MW022493	MT424687	MT424707
*D.litchicola*	BRIP 54900	*Litchichinensis*	Australia	LC041036	NA	NA	JX862539	NA
*D.litchii*	SAUCC194.22	*Litchichinensis*	China	MT822550	MT855635	MT855519	MT855747	MT855863
*D.lithocarpus*	CGMCC 3.15175	*Lithocarpusglabra*	China	KC135104	KF576235	NA	KC153095	KF576311
*D.longicolla*	FAU599	*Glycinemax*	USA	KJ590728	KJ612124	KJ659188	KJ590767	KJ610883
*D.longispora*	CBS 194.36	*Ribes*	Canada	MH855769	KC343377	KC343619	KC343861	KC344103
*D.lusitanicae*	CBS 123212	*Foeniculumvulgare*	Portugal	MH863279	KC343378	KC343620	KC343862	KC344104
*D.lutescens*	SAUCC194.36	*Chrysalidocarpuslutescens*	China	MT822564	MT855647	MT855533	MT855761	MT855877
*D.macadamiae*	CBS 146455	*Macadamia*	Australia	MN708230	NA	NA	MN696528	MN696539
*D.macintoshii*	BRIP 55064a	*Rapistrumrugosum*	Australia	KJ197289	NA	NA	KJ197251	KJ197269
*D.mahothocarpus*	CGMCC 3.15181	*Lithocarpusglabra*	China	KC153096	NA	NA	KC153087	KF576312
*D.malorum*	CAA 734	*Malusdomestica*	Portugal	KY435638	KY435658	KY435648	KY435627	KY435668
*D.masirevicii*	BRIP 54256	*Glycinemax*	Australia	KJ197277	NA	NA	KJ197238	KJ197256
*D.mayteni*	CBS 133185	*Maytenusilicifolia*	Brazil	KC343139	KC343381	KC343623	KC343865	KC344107
*D.maytenicola*	CPC 21896	*Maytenusacuminata*	South Africa	KF777157	NA	NA	NA	KF777250
*D.mediterranea*	SAUCC194.111	*Machiluspingii*	China	MT822639	MT855718	MT855606	MT855836	MT855951
*D.melastomatis*	SAUCC194.55	*Melastomamalabathricum*	China	MT822583	MT855664	MT855551	MT855780	MT855896
*D.melonis*	CBS 435.87	*Glycinesoja*	Indonesia	KC343141	KC343383	KC343625	KC343867	KC344109
*D.middletonii*	BRIP 54884e	*Rapistrumrugosum*	Australia	KJ197286	NA	NA	KJ197248	KJ197266
*D.minima*	CGMCC 3.20097	Unknown	China	MT385953	MT424722	MW022496	MT424688	MT424708
*D.minusculata*	CGMCC 3.20098	Unknown	China	MT385957	MW022475	MW022499	MT424692	MT424712
*D.miriciae*	BRIP 54736j	*Helianthusannuus*	Australia	KJ197282	NA	NA	KJ197244	KJ197262
*D.multigutullata*	CFCC 53095	*Citrusmaxima*	China	MK432645	MK442967	MK442992	MK578121	MK578048
*D.multigutullata*	CFCC 53096	*Citrusmaxima*	China	MK432646	MK442968	MK442993	MK578122	MK578049
*D.musigena*	CBS 129519	*Musa*	Australia	KC343143	KC343385	KC343267	KC343869	KC344111
*D.neoarctii*	CBS 109490	*Ambrosiatrifida*	USA	KC343145	KC343387	KC343629	KC343871	KC344113
*D.neoraonikayaporum*	MFLUCC 14-1136	*Tectonagrandis*	Thailand	KU712449	KU749356	NA	KU749369	KU743988
*D.nothofagi*	BRIP 54801	*Nothofaguscunninghamii*	Australia	JX862530	NA	NA	JX862536	KF170922
*D.novem*	CBS 127269	*Glycinemax*	Croatia	KC343155	KC343397	KC343639	KC343881	KC344123
*D.ocoteae*	CPC 26217	*Ocoteabullata*	France	KX228293	NA	NA	NA	KX228388
*D.oraccinii*	LC3166	*Camelliasinensis*	China	KP267863	NA	KP293517	KP267937	KP293443
*D.ovalispora*	ZJUD93	*Citruslimon*	China	KJ490628	NA	KJ490570	KJ490507	KJ490449
*D.ovoicicola*	CGMCC 3.17093	*Lithocarpusglabra*	China	KF576265	KF576223	NA	KF576240	KF576289
*D.oxe*	CBS 133186	*Maytenusilicifolia*	Brazil	KC343164	KC343406	KC343648	KC343890	KC344132
*D.padina*	CFCC 52590	*Padusracemosa*	China	MH121525	MH121443	MH121483	MH121567	MH121604
*D.padina*	CFCC 52591	*Padusracemosa*	China	MH121526	MH121444	MH121484	MH121568	MH121605
*D.pandanicola*	MFLUCC 17-0607	*Pandanaceae*	Thailand	MG646974	NA	NA	NA	MG646930
*D.paranensis*	CBS 133184	*Maytenusilicifolia*	Brazil	KC343171	KC343413	KC343655	KC343897	KC344139
*D.parapterocarpi*	CPC 22729	*Pterocarpusbrenanii*	Zambia	KJ869138	NA	NA	NA	KJ869248
*D.parvae*	PSCG 035	*Pyrusbretschneideri*	China	MK626920	MK691169	MK726211	MK654859	MK691249
*D.pascoei*	BRIP 54847	*Perseaamericana*	Australia	MK111097	NA	NA	JX862538	KF170924
*D.passiflorae*	CPC 19183	*Passifloraedulis*	Netherlands	JX069860	NA	NA	NA	NA
*D.passifloricola*	CPC 27480	*Passiflorafoetida*	Malaysia	KX228292	NA	KX228367	NA	KX228387
*D.penetriteum*	LC3215	*Camelliasinensis*	China	KP267879	NA	NA	KP293532	KP267953
*D.perjuncta*	CBS 109745	*Ulmusglabra*	Austria	KC343172	KC343414	KC343656	KC343898	KC344140
*D.perseae*	CBS 151.73	*Perseagratissima*	Netherlands	KC343173	KC343415	NA	NA	NA
*D.pescicola*	MFLUCC 16-0105	*Prunuspersica*	China	KU557555	KU557603	NA	KY400831	KU557579
*D.phaseolorum*	AR4203	*Phaseolusvulgaris*	USA	KJ590738	KJ612135	KJ659220	KJ590739	KJ610893
*D.phillipsii*	CAA 817	*Vacciniumcorymbosum*	Portugal	MK792305	MK883831	MK871445	MK828076	MN000351
*D.podocarpi-macrophylli*	LC6155	*Podocarpusmacrophyllus*	Japan	KX986774	KX999278	KX999246	KX999167	KX999207
*D.pometiae*	SAUCC194.72	*Pometiapinnata*	China	MT822600	MT855679	MT855568	MT855797	MT855912
***D.pseudoalnea***	**CFCC 54190**	***Alnusglutinosa***	**Netherlands**	**MZ727037**	**MZ753468**	**MZ781302**	**MZ816343**	**MZ753487**
***D.pseudoalnea***	**M2A**	***Alnusglutinosa***	**Netherlands**	**MZ727038**	**MZ753469**	**MZ753478**	**MZ816344**	**MZ753488**
*D.pseudomangiferae*	CBS 101339	*Mangiferaindica*	Dominican Republic	KC343181	KC343423	KC343665	KC343907	KC344149
*D.pseudophoenicicola*	CBS 176.77	*Mangiferaindica*	Iraq	KC343183	KC343425	KC343667	KC343909	KC344151
*D.pseudotsugae*	MFLU 15-3228	*Pseudotsugamenziesii*	Italy	KY964225	KY964138	NA	KY964181	KY964108
*D.psoraleae*	CPC 21634	*Psoraleapinnata*	South Africa	KF777158	NA	NA	KF777245	KF777251
*D.psoraleae-pinnatae*	CPC 21638	*Psoraleapinnata*	South Africa	KF777159	NA	NA	NA	KF777252
*D.pterocarpicola*	MFLUCC 10-0580a	*Pterocarpusindicus*	Thailand	JQ619887	JX197433	NA	JX275403	JX275441
*D.pungensis*	SAUCC194.112	*Elaeagnuspungens*	China	MT822640	MT855719	MT855607	MT855837	MT855952
*D.pyracanthae*	CAA483	*Pyracanthacoccinea*	Portugal	KY435635	KY435645	KY435656	KY435625	KY435666
*D.racemosae*	CPC 26646	*Euclearacemosa*	South Africa	MG600223	MG600219	MG600221	MG600225	MG600227
*D.raonikayaporum*	CBS 133182	*Spondiasmombin*	Brazil	KC343188	KC343430	KC343672	KC343914	KC344156
*D.ravennica*	MFLUCC 16-0997	*Clematisvitalba*	Italy	NA	NA	NA	MT394670	NA
*D.rhusicola*	CPC 18191	*Rhuspendulina*	South Africa	JF951146	NA	NA	NA	NA
*D.rosae*	MFLUCC 17-2658	*Rosa*	United Kingdom	MG828894	MG829273	NA	NA	MG843878
*D.rosiphthora*	COAD 2914	*Rosa*	Brazil	MT311197	MT313691	NA	MT313693	NA
*D.rossmaniae*	CAA 762	*Vacciniumcorymbosum*	Portugal	MK792290	MK883822	MK871432	MK828063	MK837914
*D.rostrata*	CFCC 50062	*Juglansmandshurica*	China	KP208847	KP208849	KP208851	KP208853	KP208855
*D.rostrata*	CFCC 50063	*Juglansmandshurica*	China	KP208848	KP208850	KP208852	KP208854	KP208856
*D.rudis*	AR3422	*Laburnumanagyroides*	Austria	KC843331	KC843146	NA	KC843090	KC843177
***D.rudis***	**CFCC 54193**	***Quercusrobur***	**Netherlands**	**MZ727039**	**MZ753470**	**MZ753479**	**MZ816345**	**MZ753489**
***D.rudis***	**M86**	***Quercusrobur***	**Netherlands**	**MZ727040**	**MZ753471**	**MZ753480**	**MZ816346**	**MZ753490**
*D.saccarata*	CBS 116311	*Protearepens*	South Africa	KC343190	KC343432	KC343674	KC343916	KC344158
*D.sackstonii*	BRIP 54669b	*Helianthusannuus*	Australia	KJ197287	NA	NA	KJ197249	KJ197267
*D.salicicola*	BRIP 54825	*Salixpurpurea*	Australia	JX862531	NA	NA	JX862537	KF170923
*D.sambucusii*	CFCC 51986	*Sambucuswilliamsii*	China	KY852495	KY852499	KY852503	KY852507	KY852511
*D.sambucusii*	CFCC 51987	*Sambucuswilliamsii*	China	KY852496	KY852500	KY852504	KY852508	KY852512
*D.schimae*	CFCC 53103	*Schimasuperba*	China	MK442640	MK442962	MK442987	MK578116	MK578043
*D.schimae*	CFCC 53104	*Schimasuperba*	China	MK442641	MK442963	MK442988	MK578117	MK578044
*D.schimae*	CFCC 53105	*Schimasuperba*	China	MK442642	MK442964	MK442989	MK578118	MK578045
*D.schini*	CBS 133181	*Schinusterebinthifolius*	Brazil	KC343191	KC343433	KC343675	KC343917	KC344159
*D.schisandrae*	CFCC 51988	*Schisandrachinensis*	China	KY852497	KY852501	KY852505	KY852509	KY852513
*D.schisandrae*	CFCC 51989	*Schisandrachinensis*	China	KY852498	KY852502	KY852506	KY852510	KY852514
*D.schoeni*	MFLU 15-1279	*Schoenusnigricans*	Italy	KY964226	KY964139	NA	KY964182	KY964109
*D.sclerotioides*	CBS 296.67	*Cucumissativus*	Netherlands	MH858974	KC343435	KC343677	KC343919	KC344161
*D.searlei*	CBS 146456	*Macadamia*	Australia	MN708231	NA	NA	NA	MN696540
*D.sennae*	CFCC 51636	*Sennabicapsularis*	China	KY203724	KY228875	NA	KY228885	KY228891
*D.sennae*	CFCC 51637	*Sennabicapsularis*	China	KY203725	KY228876	NA	KY228886	KY228892
*D.sennicola*	CFCC 51634	*Sennabicapsularis*	China	KY203722	KY228873	KY228879	KY228883	KY228889
*D.sennicola*	CFCC 51635	*Sennabicapsularis*	China	KY203723	KY228874	KY228880	KY228884	KY228890
*D.serafiniae*	BRIP 55665a	*Helianthusannuus*	Australia	KJ197274	NA	NA	KJ197236	KJ197254
*D.shaanxiensis*	CFCC 53106	*on branches of liana*	China	MK432654	MK442976	MK443001	MK578130	NA
*D.shaanxiensis*	CFCC 53107	*on branches of liana*	China	MK432655	MK432977	MK432002	MK578131	NA
*D.siamensis*	MFLUCC 10-0573a	*Dasymaschalon*	Thailand	NA	JQ619897	NA	JX275393	JX275429
***D.silvicola***	**CFCC 54191**	***Fraxinusexcelsior***	**Netherlands**	**MZ727041**	**MZ753472**	**MZ753481**	**MZ816347**	**MZ753491**
***D.silvicola***	**M79**	***Fraxinusexcelsior***	**Netherlands**	**MZ727042**	**MZ753473**	**MZ753482**	**MZ816348**	**MZ753492**
*D.sojae*	FAU635	*Glycinemax*	USA	KJ590719	KJ612116	KJ659208	KJ590762	KJ610875
*D.spartinicola*	CPC 24951	*Spartium junceμm*	Spain	KR611879	NA	KR857696	NA	KR857695
*D.spinosa*	PSCG 383	*Pyruspyrifolia*	China	MK626849	MK691129	MK726156	MK654811	MK691234
*D.sterilis*	CBS 136969	*Vacciniumcorymbosum*	Italy	KJ160579	KJ160548	MF418350	KJ160611	KJ160528
*D.stictica*	CBS 370.54	*Buxussampervirens*	Italy	KC343212	KC343454	KC343696	KC343938	KC344180
*D.subclavata*	ZJUD95	*Citrusunshiu*	China	KJ490630	NA	KJ490572	KJ490509	KJ490451
*D.subcylindrospora*	KUMCC 17-0151	Unknown	China	MG746629	NA	NA	MG746630	MG746631
*D.subellipicola*	KUMCC 17-0153	Unknown	China	MG746632	NA	NA	MG746633	MG746634
*D.subordinaria*	CBS 464.90	*Plantagolanceolata*	South Africa	KC343214	KC343456	KC343698	KC343940	KC344182
*D.taoicola*	MFLUCC 16-0117	*Prunuspersica*	China	KU557567	NA	NA	KU557636	KU557591
*D.tectonae*	MFLUCC 12-0777	*Tectonagrandis*	Thailand	KU712430	KU749345	NA	KU749359	KU743977
*D.tectonendophytica*	MFLUCC 13-0471	*Tectonagrandis*	Thailand	KU712439	KU749354	NA	KU749367	KU743986
*D.tectonigena*	MFLUCC 12-0767	*Camelliasinensis*	China	KX986782	KX999284	KX999254	KX999174	KX999214
*D.terebinthifolii*	CBS 133180	*Schinusterebinthifolius*	Brazil	KC343216	KC343458	KC343700	KC343942	KC344184
*D.ternstroemia*	CGMCC 3.15183	*Ternstroemiagymnanthera*	China	KC153098	NA	NA	KC153089	NA
*D.thunbergii*	MFLUCC 10-0576a	*Thunbergialaurifolia*	Thailand	JQ619893	JX197440	NA	JX275409	NA
*D.thunbergiicola*	MFLUCC 12-0033	*Thunbergialaurifolia*	Thailand	KP715097	NA	NA	KP715098	NA
*D.tibetensis*	CFCC 51999	*Juglandisregia*	China	MF279843	MF279888	MF279828	MF279858	MF279873
*D.tibetensis*	CFCC 52000	*Juglandisregia*	China	MF279844	MF279889	MF279829	MF279859	MF279874
*D.torilicola*	MFLUCC 17-1051	*Torilisarvensis*	Italy	KY964212	KY964127	NA	KY964168	KY964096
*D.toxica*	CBS 534.93	*Lupinusangustifolius*	Australia	KC343220	KC343462	KC343704	KC343946	KC344188
*D.tulliensis*	BRIP 62248a	*Theobromacacao*	Australia	KR936130	NA	NA	KR936133	KR936132
*D.ueckerae*	FAU656T	*Cucumismelo*	USA	KJ590726	KJ612122	KJ659215	KJ590747	KJ610881
*D.ukurunduensis*	CFCC 52592	*Acerukurunduense*	China	MH121527	MH121445	MH121485	MH121569	NA
*D.ukurunduensis*	CFCC 52593	*Acerukurunduense*	China	MH121528	MH121446	MH121486	MH121570	NA
*D.undulata*	LC6624	Unknown	China	KX986798	NA	KX999269	KX999190	KX999230
*D.unshiuensis*	ZJUD52	*Citrusunshiu*	China	KJ490587	NA	KJ490529	KJ490466	KJ490408
*D.unshiuensis*	CFCC 52594	*Caryaillinoensis*	China	MH121529	MH121447	MH121487	MH121571	MH121606
*D.unshiuensis*	CFCC 52595	*Caryaillinoensis*	China	MH121530	MH121448	MH121488	MH121572	MH121607
*D.vaccinii*	CBS 160.32	*Oxycoccusmacrocarpos*	USA	MH121502	MH121426	MH121462	MH121544	MH121584
*D.vangueriae*	CBS 137985	*Vangueriainfausta*	Zambia	KJ869137	NA	NA	NA	KJ869247
*D.vawdreyi*	BRIP 57887a	*Psidiumguajava*	Australia	KR936126	NA	NA	KR936129	KR936128
*D.velutina*	LC4421	*Neolitsea*	China	KX986790	NA	KX999261	KX999182	KX999223
*D.verniciicola*	CFCC 53109	*Verniciamontana*	China	MK573944	MK574583	MK574599	MK574619	MK574639
*D.verniciicola*	CFCC 53110	*Verniciamontana*	China	MK573945	MK574584	MK574600	MK574620	MK574640
*D.viniferae*	JZB320071	*Vitisvinifera*	China	MK341551	MK500119	NA	MK500107	MK500112
*D.virgiliae*	CMW 40748	*Virgiliaoroboides*	South Africa	KP247556	NA	NA	NA	KP247575
*D.xishuangbanica*	LC6707	*Camelliasinensis*	China	KX986783	NA	KX999255	KX999175	KX999216
*D.xunwuensis*	CFCC 53085	Unknown	China	MK432663	MK442983	MK443008	MK578137	MK578063
*D.xunwuensis*	CFCC 53086	Unknown	China	MK432664	MK442984	MK443009	MK578138	MK578064
*D.yunnanensis*	LC6168	Unknown	China	KX986796	KX999290	KX999267	KX999188	KX999228
*D.zaobaisu*	PSCG 031	*Pyrusbretschneideri*	China	MK626922	NA	MK726207	MK654855	MK691245
*Diaporthellacorylina*	CBS 121124	*Corylus*	NA	KC343004	KC343246	KC343488	KC343730	KC343972

Note: NA, not applicable. Strains in this study are marked in bold.

## Results

### Phylogenetic analyses

**Figure 1.**
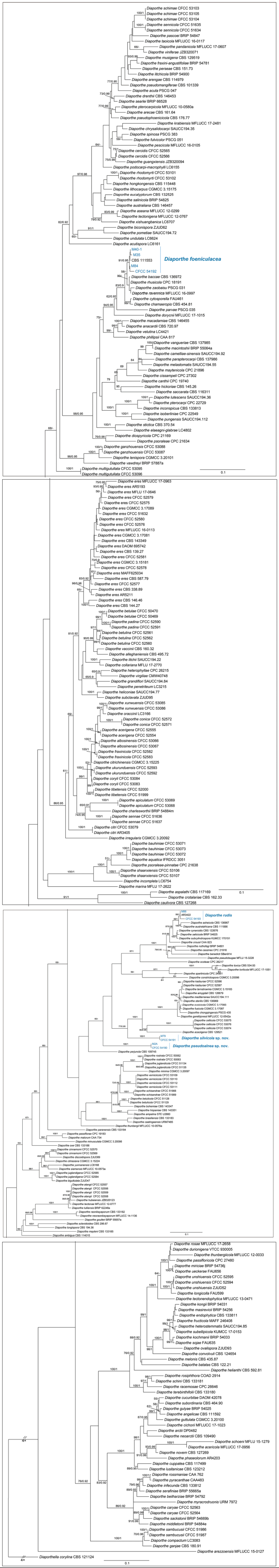
Phylogram of *Diaporthe* resulting from a maximum likelihood analysis based on a combined matrix of ITS, *cal*, *his3*, *tef1* and *tub2*. Numbers above the branches indicate ML bootstraps (left, ML BS ≥ 50 %) and Bayesian Posterior Probabilities (right, BPP ≥ 0.75). The tree is rooted with *Diaporthellacorylina*. Isolates from present study are marked in blue.

The five-gene sequence dataset (ITS, *cal*, *his3*, *tef1* and *tub2*) was analysed to infer the interspecific relationships within *Diaporthe*. The dataset consisted of 307 sequences including one outgroup taxon, *Diaporthellacorylina* (CBS 121124). A total of 2649 characters including gaps (516 for ITS, 576 for *cal*, 526 for *his3*, 507 for *tef1* and 524 for *tub2*) were included in the phylogenetic analysis. Of these characters, 844 were constant, 318 were variable and parsimony-uninformative, and 1487 were parsimony-informative. The topologies resulting from ML and BI analyses of the concatenated dataset were congruent (Fig. 1). Isolates from the present study formed four individual clades representing four species of *Diaporthe*, of which isolates CFCC 54192, M35, M40-1 and M84 from *Quercusrobur* represent *D.foeniculacea*, while CFCC 54193 and M86 from *Q.robur* represent *D.rudis*. CFCC 54191 and M79 from *Fraxinusexcelsior* and CFCC 54190 and M2A from *Alnusglutinosa* represent two new species which are here described as *D.silvicola* and *D.pseudoalnea*, respectively.

## Taxonomy

### ﻿ Diaporthe pseudoalnea

Taxon classificationFungiDiaporthalesDiaporthaceae

N. Jiang sp. nov.

ABC17B34-3B5B-5E2F-A337-152C8D79C8D9

840714

Fig. 2

#### Etymology.

With reference to *D.alnea*, which was described from the same host genus, *Alnus*.

#### Description.

Conidiomata pycnidial, discoid, immersed in bark, scattered, erumpent through the bark surface, with a solitary locule. Locule 800–1250 μm diam., undivided. ﻿Conidiophores 22–68.5 × 1.5–3 μm (av. = 39.8 × 2.2 μm, n = 50), cylindrical, attenuate towards the apex, hyaline, slightly brown at base, phialidic, unbranched, straight or slightly curved. Alpha conidia (5.8–)7.1–8.9(–11.2) × (1.5–)1.8–2.2(–2.7) μm (av. = 7.9 × 2.0 μm, n = 50), L/W = 3.2–4.7 (av. = 3.8, n = 50), hyaline, aseptate, subcylindrical with a nearly rounded apex, multi-guttulate, sometimes acute at both ends. Beta conidia not observed.

**Figure 2.**
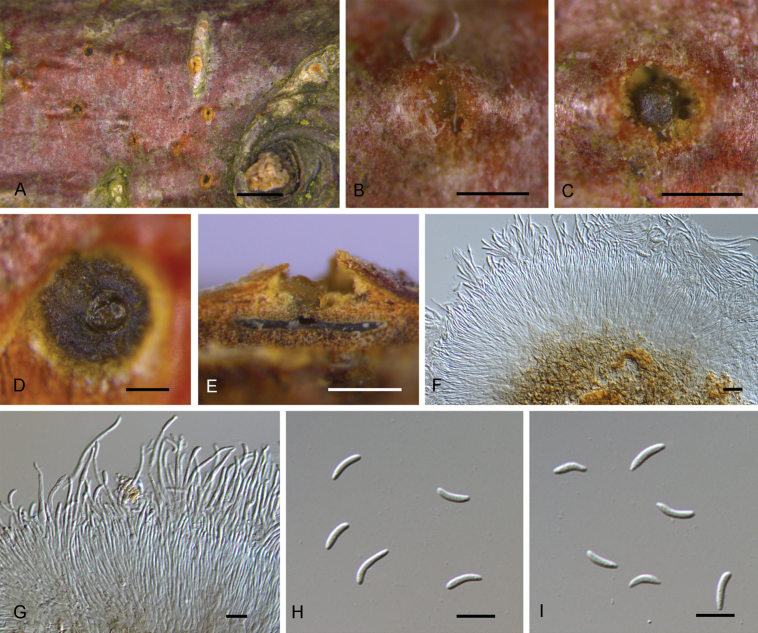
*Diaporthepseudoalnea* from *Alnusglutinosa***A–C** habit of conidiomata on branches **D** transverse section of conidiomata **E** longitudinal section through conidiomata **F, G** conidiophores and conidia **H, I** conidia. Scale bars: 2 mm (**A**), 500 μm (**B, C, E**), 200 μm (**D**), 10 μm (**F–I**).

#### Culture characters.

Colonies are initially white with fluffy aerial mycelium, becoming dirty white after 2 weeks, and conidiomata are randomly distributed with orange conidial drops oozing out of the ostioles.

#### Specimens examined.

NETHERLANDS. Utrecht City, on branches of *Alnusglutinosa*, 5°11’32” E, 52°05’22” N, 8 Apr. 2019, *N. Jiang* (holotype CAF800005 = JNH0001; ex-type living culture: CFCC 54190; other living culture: M2A).

#### Notes.

*Diaporthenivosa* and *D.alnea* were recorded from the host genus *Alnus*. Udayanga et al. (2014) investigated the lectotype of *Diaporthenivosa* and revealed it as a *Melanconis* species based on a well-developed ectostromata and the ascospores characteristics, and Jaklitsch and Voglmayr (2020) treated it as a synonym of Melanconismarginalisssp.marginalis. *D.alnea* has been reported from the Czech Republic, Germany, the Netherlands and the USA, and both sexual and asexual morphs have been described (Udayanga et al. 2014). However, applying the GCPSR principle, *D.alnea* has recently been considered to be a synonym of *Diaportheeres* (Hilário et al. 2021), which has also been confirmed in our analyses where the ex-epitype isolate CBS 146.46 of *D.alnea* is placed within the *D.eres* clade (Fig. 1). *Diaporthepseudoalnea* morphologically differs from *D.alnea* (now *D.eres*) by ﻿its longer conidiophores (22–68.5 × 1.5–3 μm in *D.pseudoalnea* vs. 9–16 × 1–2 μm in *D.alnea*; Udayanga et al. 2014). In our multi-gene analyses, *D.pseudoalnea* forms a distinct phylogenetic lineage which is placed remotely from the isolate CBS 146.46 of *D.alnea* (Fig. 1).

### Diaporthe silvicola

Taxon classificationFungiDiaporthalesDiaporthaceae

N. Jiang sp. nov.

A563ADB6-35CD-5A06-9D98-8A27FCC9D26A

840715

Fig. 3

#### Etymology.

Name from “*silva*” = forest and “-*cola*” = inhabiting; with reference to its woody host.

#### Description.

Conidiomata pycnidial, conical, immersed in bark, scattered, erumpent through the bark surface, with a solitary locule. Locule 450–700 μm diam., undivided. Conidiophores 6.5–25 × 1.5–4 μm (av. = 15.4 × 2.4 μm, n = 50), cylindrical, attenuate towards the apex, hyaline, slightly brown, phialidic, unbranched, slightly curved. ﻿Alpha conidia (9.2–)10.1–12.3(–13.5) × (3.8–)4.2–4.9(–5.2) μm (av. = 11.5 × 4.5 μm, n = 50), L/W = 2.0–3.2 (av. = 2.5, n = 50), hyaline, aseptate, fusiform to oval, multi-guttulate, acute at both ends. Beta conidia not observed.

**Figure 3.**
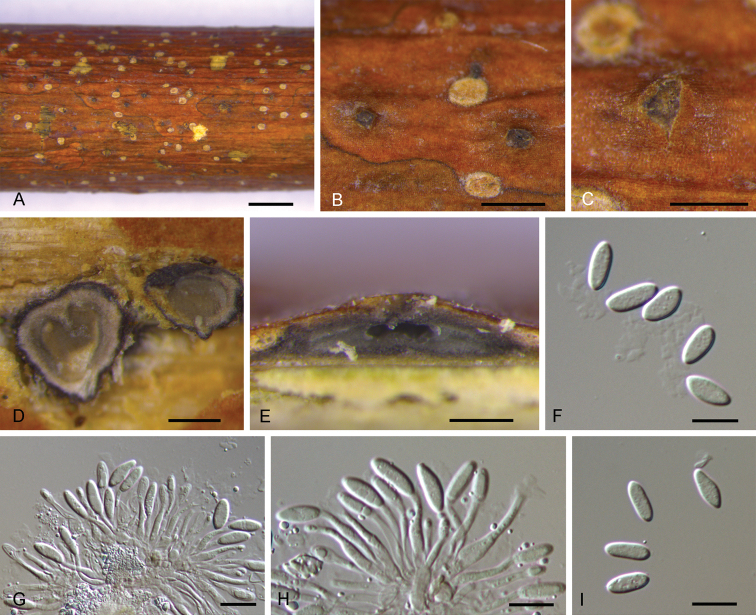
*Diaporthesilvicola* from *Fraxinusexcelsior***A–C** habit of conidiomata on branches **D** transverse section of conidiomata **E** longitudinal section through conidiomata **F, I** conidia **G, H** conidiophores and conidia. Scale bars: 2 mm (**A**), 1 mm (**B**), 500 μm (**C**), 200 μm (**D, E**), 10 μm (**F–I**).

#### Culture characters.

Colonies are initially white, aerial mycelium turning grey at edges of plate, yellowish pigmentation developing in centre, conidiomata not produced until 2 weeks.

#### Specimens examined.

﻿NETHERLANDS. Utrecht City, on branches of *Fraxinusexcelsior* in the forest ecosystem, 5°10’36” E, 52°05’32” N, 6 Jun. 2019, *N. Jiang* (holotype CAF800006 = JNH0002; ex-type living culture: CFCC 54191; other living culture: M79).

#### Notes.

*Diaporthefraxini-angustifoliae* was reported from Fraxinusangustifoliasubsp.oxycarpa cv. Claret Ash in Australia (Tan et al. 2013). *D.fraxinicola* was described from *Fraxinuschinensis* in China (Yang et al. 2018). However, *D.silvicola* from *Fraxinusexcelsior* in Netherlands differs from *D.fraxini-angustifoliae* and *D.fraxinicola* by obviously larger alpha conidia (9.2–13.5 × 3.8–5.2 μm in *D.silvicola* vs. 4–10 × 2–3 μm in *D.fraxini-angustifoliae* vs. 7–10 × 2.9–3.2 μm in *D.fraxinicola*; Tan et al. 2013; Yang et al. 2018).

## Discussion

In this study, branch-inhabiting *Diaporthe* species were sampled from *Alnusglutinosa*, *Fraxinusexcelsior* and *Quercusrobur* in Utrecht, the Netherlands. Ten *Diaporthe* isolates were obtained and identified based on five combined loci (ITS, *cal*, *his3*, *tef1* and *tub2*), as well as morphological characters from the natural substrates. The phylogenetic and morphological analyses revealed *Diaporthepseudoalnea* sp. nov. from *Alnusglutinosa*, *Diaporthesilvicola* sp. nov. from *Fraxinusexcelsior*, and *D.foeniculacea* and *D.rudis* from *Quercusrobur*.

Phylogenetic analyses were conducted based on a combined DNA sequence matrix of five loci (ITS, *cal*, *his3*, *tef1* and *tub2*) reported as useful markers to distinguish species of *Diaporthe* (Udayanga et al. 2014, 2015; Guarnaccia et al. 2017, 2018a, 2018b; Tibpromma et al. 2018; Yang et al. 2020; Dissanayake et al. 2020; Huang et al. 2021; Sun et al. 2021, Wang et al. 2021). The two novel species in this study can be distinguished from the other known species by all genes studied, but most effectively by *cal*, *his3*, *tef1* and *tub2*. The multi-locus phylogenetic analysis grouped the isolates in two new clades, which support the introduction of the new species.

The utility of host association for *Diaporthe* species identification is limited because several species have wide host ranges (e.g., *D.ere* inhabits 282 different hosts; *D.rudis* inhabits 44 different hosts), and multiple *Diaporthe* species can infect a single host (e.g., nineteen *Diaporthe* species are associated with pear cankers in China) (Guo et al. 2020; Farr and Rossman 2021). Thus, a polyphasic approach of morphological, cultural, ecological and molecular data to identify *Diaporthe* samples or to introduce new species is essential.

## Supplementary Material

XML Treatment for Diaporthe pseudoalnea

XML Treatment for Diaporthe silvicola
